# Generating trunk neural crest from human pluripotent stem cells

**DOI:** 10.1038/srep19727

**Published:** 2016-01-27

**Authors:** Miller Huang, Matthew L. Miller, Lauren K. McHenry, Tina Zheng, Qiqi Zhen, Shirin Ilkhanizadeh, Bruce R. Conklin, Marianne E. Bronner, William A. Weiss

**Affiliations:** 1 Department of Neurology and the Helen Diller Family Comprehensive Cancer Center, University of California, San Francisco, CA 94158 USA; 2 Departments of Pediatrics, Neurosurgery and Brain Tumor Research Center, University of California, San Francisco, CA 94158 USA; 3 Gladstone Institute of Cardiovascular Disease, San Francisco, CA 94158 USA; 4 Departments of Medicine and Cellular and Molecular Pharmacology, University of California, San Francisco, San Francisco, California 94143, USA; 5 Division of Biology and Biological Engineering, California Institute of Technology, Pasadena, California 91125, USA

## Abstract

Neural crest cells (NCC) are stem cells that generate different lineages, including neuroendocrine, melanocytic, cartilage, and bone. The differentiation potential of NCC varies according to the level from which cells emerge along the neural tube. For example, only anterior “cranial” NCC form craniofacial bone, whereas solely posterior “trunk” NCC contribute to sympathoadrenal cells. Importantly, the isolation of human fetal NCC carries ethical and scientific challenges, as NCC induction typically occur before pregnancy is detectable. As a result, current knowledge of NCC biology derives primarily from non-human organisms. Important differences between human and non-human NCC, such as expression of HNK1 in human but not mouse NCC, suggest a need to study human NCC directly. Here, we demonstrate that current protocols to differentiate human pluripotent stem cells (PSC) to NCC are biased toward cranial NCC. Addition of retinoic acid drove trunk-related markers and *HOX* genes characteristic of a posterior identity. Subsequent treatment with bone morphogenetic proteins (BMPs) enhanced differentiation to sympathoadrenal cells. Our approach provides methodology for detailed studies of human NCC, and clarifies roles for retinoids and BMPs in the differentiation of human PSC to trunk NCC and to sympathoadrenal lineages.

Embryonic stem cells (ESCs) in the epiblast become progressively specified during development, initially into germ layers that are then further subdivided. The outermost ectodermal layer contains precursors for neural and non-neural ectoderm^1^. During neurulation, the neural ectoderm becomes a neuroepithelium that will form the central nervous system (CNS). Neural crest precursors initially are contained within this neuroepithelium, but subsequently undergo an epithelial-to-mesenchymal transition, generating neural crest cells (NCC) which delaminate from the neural tube and migrate to distant locations throughout the body. NCC are multipotent and contribute to a wide range of distinct lineages including chondrocytes, osteocytes, melanocytes, sensory neurons, smooth muscle cells, Schwann cells, chromaffin cells and sympathetic neurons^1^,^2^. Not all NCC are alike, with distinct populations existing along the neural axis. For instance, NCC arising from cranial and trunk levels can differentiate into neurons, glial cells, and pigment cells. Only cells from cranial axial level contribute to bone and cartilage of the face, whereas trunk NCC lack the ability to do so, even when grafted to the head. Conversely, sympathoadrenal (SA) cells normally arise only from trunk NCC.

Current knowledge about NCC development and biology has come primarily from studies in chick, zebrafish and mouse^1^,^2^,^3^. This work uncovered markers expressed by NCC, including *TFAP2A*, *FOXD3*, *B3GAT1* (HNK1), *NGFR* (p75), *SOX9* and *SOX10*^4^,^5^,^6^,^7^,^8^,^9^,^10^ and were validated subsequently in humans^11^,^12^. While these genes are generally expressed in NCC at all axial levels, distinct axial level specific enhancers drive the expression of some markers. For example, *SOX10* is driven by the SOX10E2 enhancer in cranial NCC but by SOX10E1 in trunk NCC^13^. Similarly, a cranial-specific NC1 enhancer drives *FOXD3* expression in the cranial neural crest, but not the trunk NCC^14^. There are also axial-level selective neural crest transcription factors. For example, ETS1 is a cranial NCC specific transcription factor^15^,^16^ and a direct input into cranial NCC enhancers Sox10E2 and NC1 for *FOXD3*. While many cranial NCC markers are known^17^, specific markers of trunk NCC remain poorly characterized.

The SA lineage is uniquely derived from trunk NCC. Differentiation of trunk NCC to SA cells occurs when trunk NCC migrate ventrally adjacent to the dorsal aorta, which secretes bone morphogenic proteins (BMPs) to trigger upregulation of early SA markers such as PHOX2B^18^,^19^,^20^. SA cells are multipotent progenitor cells that give rise to both sympathetic neurons and neuroendocrine cells such as chromaffin cells of the adrenal medulla. These cells express catecholaminergic synthetic enzymes, including tyrosine hydroxylase (TH) and dopamine beta hydroxylase (DBH). Defects in adrenal medulla function can promote hypotension, while transformation in the adrenal medulla can lead to pheochromocytoma or neuroblastoma^21^,^22^. Although non-human SA cells have been used to model and study these diseases^22^,^23^, differences exist between human and non-human cell based systems^24^,^25^. Similarly, discrepancies in development between human and non-human NCC, and between different non-human NCC species have also been identified^5^,^11^,^12^. Therefore, generation of a renewable source of human cell-based trunk NCC provides a reliable and representative system to study human development and disease. However, in addition to ethical issues surrounding isolation of human fetal tissue, obtaining human fetal NCC is particularly difficult because induction and migration of NCC typically occurs before pregnancy is detected^26^. Thus, differentiation of human pluripotent stem cells (PSC) to trunk NCC represents the most feasible method of obtaining human trunk NCC.

Early protocols to convert human PSC to NCC employed a dual SMAD inhibition strategy^27^,^28^ blocking TGF-β and BMP signaling. Another NCC differentiation protocol combined inhibition of GSK3β (activation of WNT pathway) with TGF-β blockade, but did not find increased efficiency with BMP inhibition^29^. In contrast, a more recent NCC differentiation protocol also included WNT activation and required transient BMP inhibition to induce optimal expression of the NCC marker *SOX10*^30^. Thus, the role of BMP signaling in human PSC to NCC differentiation is still unresolved, and the subtype of NCC produced by these methods is unclear.

Here, we evaluated the effect of BMP inhibition on the ability to differentiate human PSC to NCC, and investigated the subtype of NCC produced. In our hands, NCC derived from a protocol that did not manipulate BMP signaling suppressed CNS markers to a greater extent than a protocol that featured transient inhibition of BMP. NCC produced were cranial in character, expressing high levels of the cranial NCC marker *ETS1*, and low levels of trunk NCC progenitor marker, *PHOX2B*. By treating cells with retinoic acid (RA) within a narrow 2-3 day window, we drove specification more posteriorly towards trunk NCC, identifying several *HOX* genes that were upregulated in trunk but not cranial NCC. These trunk NCC could differentiate spontaneously and expressed markers of melanoblasts and SA cells. Addition of BMP further stimulated expression of SA markers, including the catecholamine synthesizing enzyme TH. Our results clarify the role of BMP signaling in NCC and SA differentiation while providing robust methodology to generate a renewable source of human trunk NCC and SA progenitor cells.

## Results

### Blockade of BMP signaling is dispensable for NCC differentiation and promotes expression of CNS-related markers

**Figure 1:**
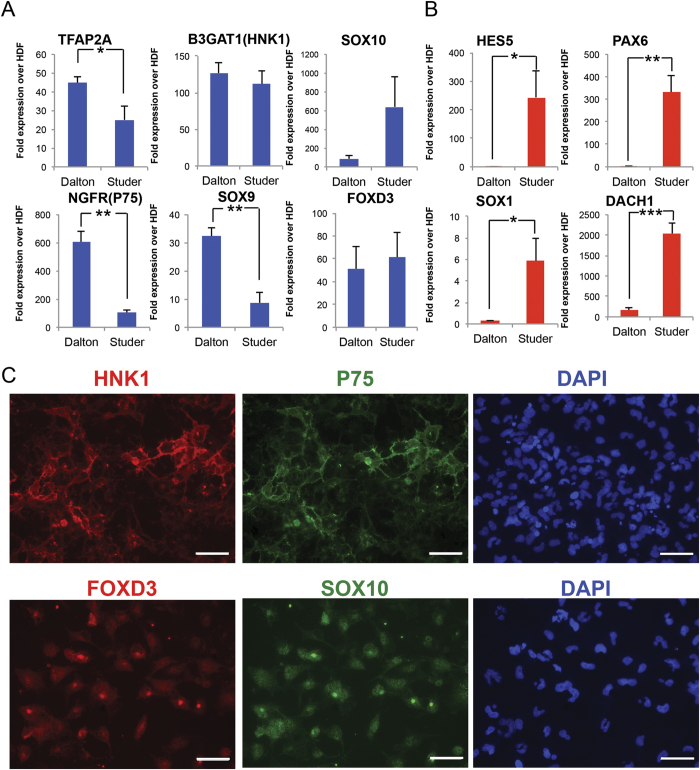
Blockade of BMP signaling is not required for NCC differentiation and promotes upregulation of CNS-related markers. (**A,B**) H1 ESC were differentiated toward NCC via protocols published by the Dalton or Studer lab. At the end of each protocol, expression of NCC markers (**A**) or CNS-related markers (**B**) was analyzed using RT-qPCR and compared against the expression of these markers in human dermal fibroblasts (HDF). Dalton protocol showed higher levels of NCC markers, while suppressing CNS-related markers compared to the Studer protocol. (**C**) Immunofluorescence analysis of Dalton protocol-derived NCC for NCC markers HNK1/p75 (top) and FOXD3/SOX10 (bottom). *p < 0.05, **p < 0.01, ***p < 0.001. Scale bars represent 50 μm.

To identify a differentiation protocol for trunk NCC, we first compared protocols published by the Dalton and Studer labs, which differed by the absence or presence of BMP inhibition (LDN193189), respectively^30^,^31^. We differentiated an ESC (H1) and iPSC (WTC) line towards NCC using both protocols. To maintain consistency, we utilized the same GSK3β inhibitor and dose (CHIR99021, 3 uM) and used the TGF-β inhibitor, SB431542 at 10 uM, as we saw comparable expression of NCC markers using 10 uM versus 20 uM of SB431542 (not shown), the original concentration used in the Dalton protocol^31^. Lastly, we stopped both protocols at Day 11 to match the length of time that cells were treated with SMAD and GSK3β inhibitor(s). NCC markers *TFAP2A*, *FOXD3*, *B3GAT1*(HNK1), *NGFR* (p75), *SOX9* and *SOX10* showed upregulation using both protocols, though the protocol without BMP inhibition showed higher expression for most markers (Fig. 1A, Supplementary Figure S1). Human dermal fibroblasts (HDF) were used as the negative control for the NCC markers because expression of all NCC markers was lower in HDFs than in PSCs (data not shown). To confirm that these protocols led specifically to NCC, we next evaluated expression of CNS-related markers that are generally downregulated in NCC (*HES5*, *PAX6*, *DACH1*, *SOX1*). BMP inhibition was associated with increased expression of these CNS markers (Fig. 1B, Supplementary Figure S1). Immunofluorescence analysis supported that NCC produced without BMP inhibition were positive for NCC markers (e.g. HNK1, p75, SOX10, FOXD3) (Fig. 1C).

To directly evaluate the role of BMP signaling in NCC and CNS marker expression, we compared the original Dalton protocol (SB431542 + CHIR99021 = “Protocol #1”) with a protocol modified by addition of the BMP inhibitor throughout (SB431542 + CHIR99021 + LDN193189 = “Protocol #2”) or modified using a timeline of inhibitor addition/withdrawl similar to the Studer protocol (“Protocol #3”) (Supplementary Figure S2). Both H1 and WTC cells exhibited highest expression of most NCC markers under Protocol #1 (Supplementary Figure S2). Addition of the BMP inhibitor LDN193189 (both Protocol #2 and #3) again increased the expression of CNS markers that should be negative in NCC, suggesting both that BMP inhibition actually promoted expression of CNS-related markers, and that differentiation of NCC did not require BMP inhibition (Supplementary Figure S2).

### Retinoic acid promotes specification of trunk NCC

**Figure 2:**
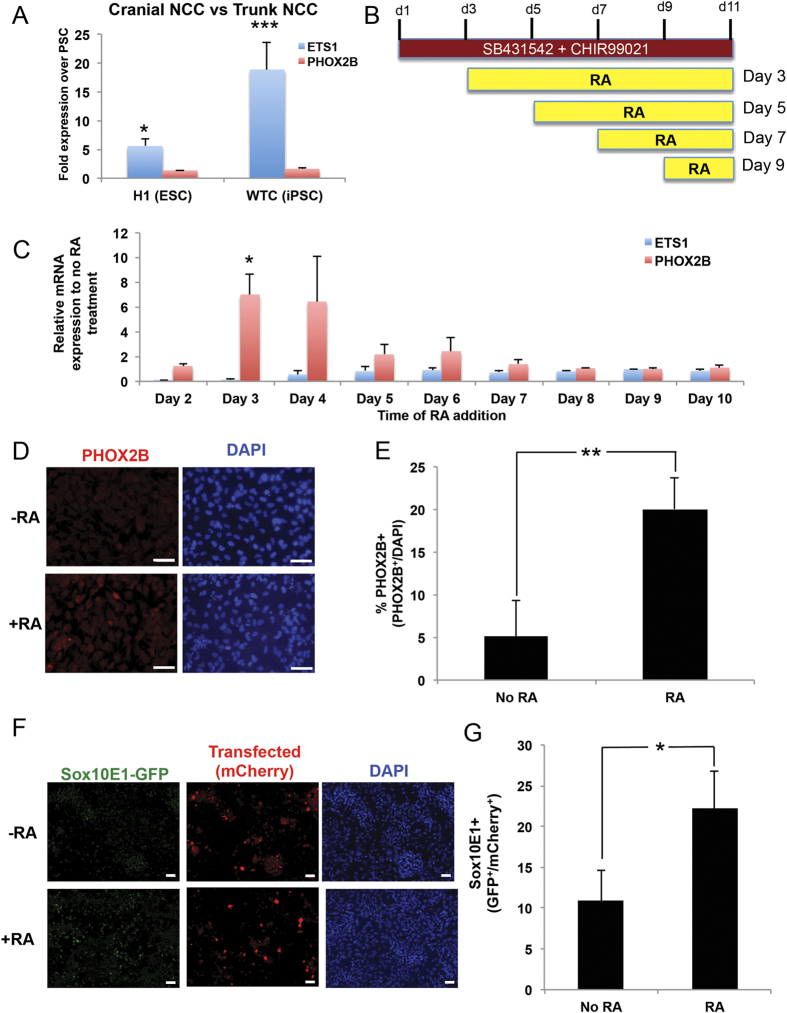
Retinoic acid promotes specification of trunk NCCs. (**A**) RT-qPCR analysis of cranial (*ETS1*) and trunk (*PHOX2B*) NCC markers from H1 and WTC differentiated via Dalton protocol. **(B)** Schematic detailing the timing of retinoic acid (RA) treatment during NCC differentiation. **(C)** RT-qPCR analysis for cranial (*ETS1*) and trunk (*PHOX2B*) markers from H1 cells differentiated with RA added starting at each day indicated, demonstrating that RA addition at Day 3 or 4 resulted in maximal upregulation of trunk (*PHOX2B*) and suppression of cranial (*ETS1*) markers. **(D)** NCC treated with or without RA were stained for PHOX2B and DAPI. **(E)** Percentage of PHOX2B^+^ cells (PHOX2B^+^ /DAPI) was quantified using ImageJ. **(F,G)** NCC differentiated without or with RA (@ day 3) were electroporated with a constitutive expressing mCherry plasmid and a SOX10 E1-GFP reporter activated specifically in trunk NCC. After 72 hours, cells were analyzed for GFP expression via immunofluorescence **(F)** and quantified using ImageJ (GFP^ + ^/mCherry^+^) **(G)** *p < 0.05, **p < 0.01. Scale bars represent 50 μm.

To clarify subtypes of NCC produced, we analyzed *ETS1*, a marker for cranial NCC and *PHOX2B*, a marker for the trunk NCC progenitor SA cells. NCC derived from Protocol #1 demonstrated robust expression of *ETS1* (cranial NCC), with no change in expression of *PHOX2B* (trunk NCC) over PSC (Fig. 2A). Thus, Protocol #1 led to formation of the cranial subtype of NCC.

To drive posterior trunk NCC character, we evaluated retinoic acid (RA), which can push cells toward a posterior fate associated with upregulation of posterior patterning *HOX* genes^32^,^33^. We added RA at different time points and analyzed expression of *ETS1* (cranial) and *PHOX2B* (trunk) (Fig. 2B). Addition of RA between days 3-4 maximally upregulated expression of *PHOX2B* and simultaneously suppressed expression of *ETS1* mRNA (Fig. 2C, Supplementary Figure S3). To evaluate cell-to-cell differences in gene expression, we performed immunofluorescence analysis. RA-treated NCC increased PHOX2B protein levels in approximately 25% of the cell population compared to non-RA treated NCC (Fig. 2D,E), indicating that RA treatment results in a heterogeneous population of trunk NCC.

In addition to the differential expression of *ETS1* and *PHOX2B*, cranial and trunk NCC also differ in the enhancer elements used to transcribe *SOX10*^13^. To assess how RA influences trunk NCC enhancer usage, we co-electroporated an mCherry plasmid (control) and a SOX10E1-GFP plasmid (marker of trunk NCC) in NCC differentiated without or with RA addition at Day 3. While mCherry was visible in both cell types, GFP was more prominent in RA treated cells (p < 0.05) (Fig. 2F,G). Thus, RA promotes specification of trunk NCC.

### RA treatment upregulates expression of *HOX* genes and alters the differentiation potential of NCC

**Figure 3:**
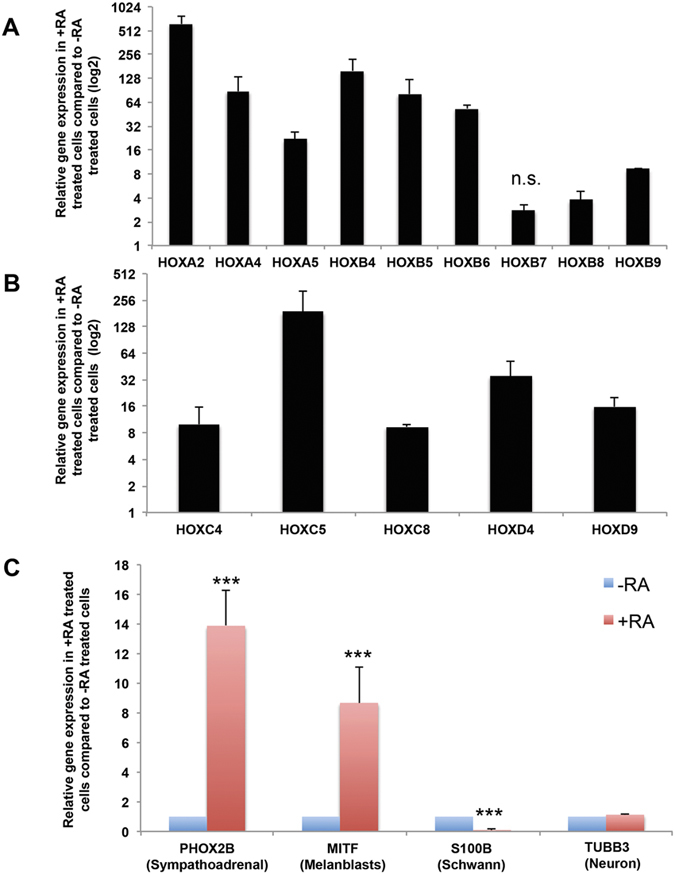
RA treatment upregulates multiple *HOX* genes and alters the differentiation potential of NCC. (**A,B**) Trunk NCC were analyzed via RT-qPCR for **(A)** *HOXA, HOXB*, (**B)** *HOXC* and *HOXD* genes previously described to be detected in trunk NCC and were found to be upregulated compared to cranial NCC (not treated with RA). All genes are expressed at statistically significant levels (p < 0.05) in trunk NCC compared to cranial NCC except for *HOXB7* (n.s. = not significant) **(C)** Trunk NCC were compared against cranial NCC for NCC progenitor markers *PHOX2B* (sympathoadrenal), *MITF* (melanoblasts), *S100β* (Schwann cells), and *TUBB3* (neurons). ***p < 0.001.

Many NCC markers are not specific to either cranial or trunk NCC^11^. However, since trunk NCC are more posterior than cranial NCC, trunk NCC express *HOX* genes whereas mesencephalic NCC do not^34^,^35^. In fact, only hindbrain NCC emerging from rhombomere 4 and further caudally express anterior *HOX* genes. *HOX* genes previously detected in trunk NCC include *HOXA2, HOXA4, HOXA5, HOXB4-9, HOXC4, HOXC5, HOXC8, HOXD4* and *HOXD9*^36^,^37^,^38^,^39^,^40^. RT-qPCR analysis of each of these *HOX* genes revealed upregulation in response to RA treatment (all at statistically significant levels (p < 0.05), except for *HOXB7*) (Fig. 3A,B).

To determine whether RA treatment alters the differentiation potential of resulting NCC, we analyzed NCC derived with or without RA treatment for expression of genes of different NCC progenitor subtypes: *PHOX2B* for SA cells, *MITF* for melanoblasts, *S100β* for Schwann cells and *TUBB3* for neurons. Addition of RA increased the expression levels of *PHOX2B* and *MITF*, decreased *S100β*, and did not have an effect on *TUBB3* compared to non-RA treated cells (Fig. 3C). Thus, RA treatment alters the gene expression profile and differentiation potential of NCC.

### BMP signaling promotes SA differentiation of trunk NCC

**Figure 4:**
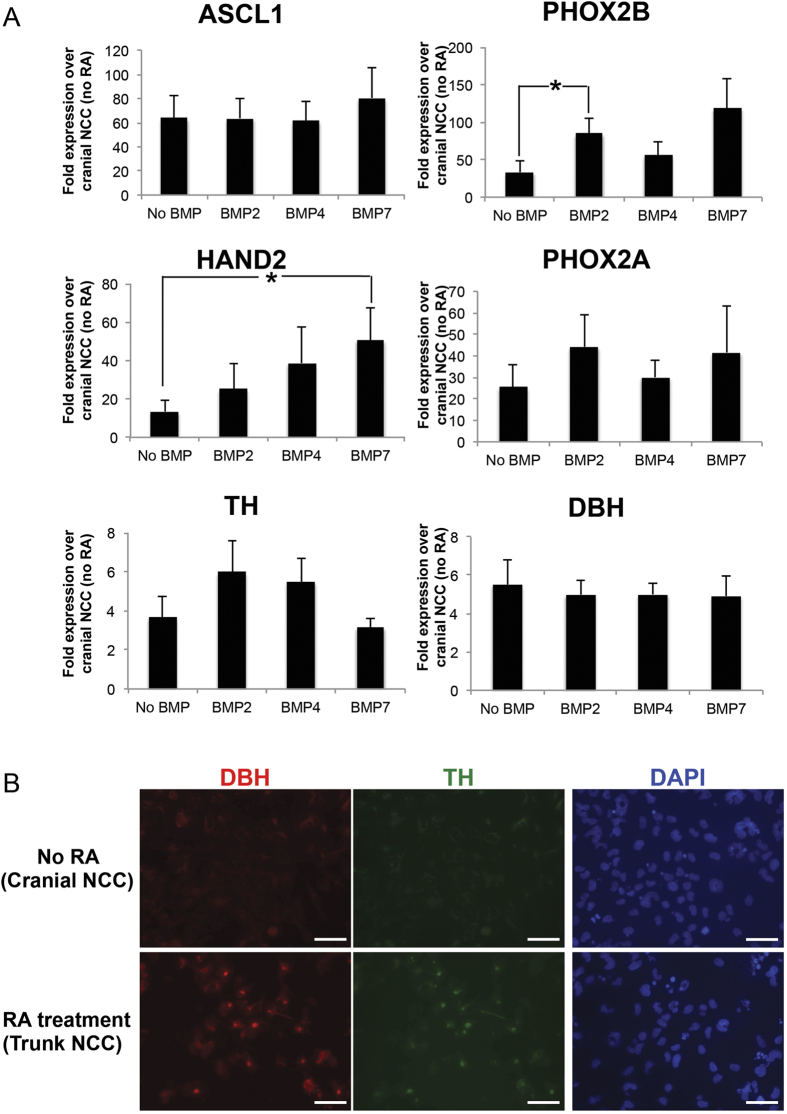
BMP signaling stimulates expression of SA makers. (**A**) Trunk NCC were treated without or with BMP-2, -4 or -7 for 2 weeks and analyzed via RT-qPCR for SA markers (*ASCL1*, *PHOX2B*, *HAND2*, *PHOX2A*, *TH*, *DBH*), which showed upregulation compared to cranial NCC. (**B**) Expression of late SA markers TH and DBH was confirmed via immunofluorescence. *p < 0.05. Scale bars represent 50 μm.

During development, trunk NCC migrate to the dorsal aorta and are exposed to BMPs which stimulate differentiation toward SA cells^41^. ASCL1 and PHOX2B are expressed initially, and subsequently upregulate expression of PHOX2A and HAND2, which in-turn drive transcription of the catecholamine synthetic enzymes TH and DBH^19^,^42^. To confirm the potential for PSC-derived trunk NCC to differentiate along the SA lineage, we therefore differentiated RA-derived trunk NCC toward late stage SA cells. After differentiating human PSC to trunk NCC, we treated the trunk NCC with BMP-2, BMP-4 or BMP-7 for 2 weeks. Different BMP ligands elevated SA markers to varying degrees (e.g. BMP-7 stimulated highest expression of *HAND2* while BMP-2 increased levels of *PHOX2B* the most) (Fig. 4A). Although *TH* was the only late stage SA marker upregulated in response to BMP treatment, both TH and DBH increased spontaneously in RA-treated NCC over the 2 weeks compared to non-RA treated NCC, as observed via immunofluorescence (Fig. 4B). These data confirm that our PSC-derived trunk NCC can produce the trunk NCC progenitor SA lineage and is responsive to BMP treatment.

## Discussion

Initial protocols to differentiate human PSC to both neuroepithelial and NCC utilized dual SMAD inhibition by blocking TGF-β (via SB431542) and BMP signaling (via Noggin or LDN-193189)^27^,^28^,^43^,^44^. Two recently described NCC differentiation protocols included activation of WNT signaling, which led to more efficient generation of NCC^29^,^30^. One key difference between these protocols was the use of BMP inhibition to induce NCC. While activation of BMP signaling can suppress differentiation to NCC^29^,^45^, developmental studies in frogs, birds, and mice suggested that inhibiting BMP signaling was not required for NCC induction^46^,^47^,^48^. In our experiments, constant or transient inhibition of BMP signaling did not drive expression of NCC markers, but did increase expression of CNS-related markers (Fig. 1, Supplementary Figure S1). Thus, inhibition of TGF-β and activation of WNT signaling were sufficient to induce expression of NCC but not neuroepithelial markers, while blocking BMP signaling promoted expression of CNS-related markers.

The utility of human PSCs is dependent on protocols to differentiate PSC towards desired cell types. Current protocols to differentiate human PSCs to NCC can generate peripheral neurons, chondrocytes, osteocytes, smooth muscle, Schwann cells, and melanocytes, but have not produced trunk NCC-derived SA cells^27^,^30^,^31^,^49^. Might these protocols bias toward cranial NCC? Indeed, we found that, in the absence of caudalization factors such as RA, current protocols bias toward cranial NCC based on upregulation of cranial specific NCC marker *ETS1* (Fig. 2A). We demonstrate that treatment with RA during NCC differentiation promoted a more caudal trunk NCC subtype based on several criteria: [1] Upregulation of the trunk NCC progenitor marker, *PHOX2B*, and downregulation of the cranial marker *ETS1* (Fig. 2C), [2] Increased levels of E1-enhancer activity on *SOX10*, which is specific for trunk NCC (Fig. 2F, 2G), [3] Expression of posterior *HOX* genes (Fig. 3A,B), and [4] Ability to differentiate and express late stage SA markers TH and DBH, which are unique to trunk NCC (Fig. 4B). Thus, our data demonstrates for the first time, to our knowledge, the ability to generate human trunk NCC from human PSC.

In addition to generating trunk-like NCC, RA treatment also alters the gene expression of different NCC progenitor markers, suggesting a shift in bias toward differentiating to particular cell lineages. Our RA-treated NCC demonstrates a stronger capacity to differentiate toward not only trunk specific SA cells, but also to melanoblasts (Fig. 3C). These trunk NCC also demonstrated decreased expression of the Schwann cell marker, *S100β*, an observation consistent with a previous report that NCC-derived Schwann cells can subsequently differentiate to melanoblasts^50^. The apparent differences in differentiation potential between non-RA treated and RA treated NCC could be the result of differential *HOX* gene expression profiles, as *HOX* genes serve not only as indicators of where NCC arise along the neuraxis, but also have functional roles in differentiation. For instance, expression of HOXA2 has been linked to blockade of chondrogenesis in cranial NCC^38^,^51^, while overexpression of HOXB8 can stimulate expression of SA markers *TH* and *DBH* in avian NCC^36^. Therefore, *HOX* genes play a significant role in determining the differentiation capacity of NCC. Our trunk NCC derived from human PSC expressed *HOX* genes that were previously detected in trunk NCC of other species (Fig. 3A,B).

RA induction of distinct *HOX* genes suggests this approach may be adapted to produce other NCC subtypes such as vagal and sacral NCC, by adjusting the concentration of RA and/or by modification/addition of the concentration of CHIR99021, Fibroblast Growth Factor (FGF) or Growth Differentiation Factor (GDF), which alter the profile of *HOX* gene expression^33^. Addition of these other factors could also enhance the efficiency of generating trunk NCC, as treatment with RA resulted in a heterogeneous population, with ~25% of cells developing trunk-like characteristics (Fig. 2D,E). These human PSC-based models of different NCC subtypes represent tools to distinguish among NCC subtypes, and provide a human-relevant model to investigate NCC biology and to compare with findings in other organisms. One notable difference between human NCC and mouse NCC is the expression of HNK1 specifically in human, but not mouse NCC^5^. Thus, while non-human model organisms have largely informed NCC biology, the NCC in model organisms may differ from human NCC.

Although BMP stimulation has been shown to block differentiation of human PSC to NCC, BMP treatment has also been implicated in differentiation of trunk NCC to SA cells, as *BMP-2/4/7* mRNA are expressed in the wall of the dorsal aorta, a location to which trunk NCC migrate to eventually differentiate toward SA cells^52^,^53^,^54^. Supporting the importance of BMP signaling in SA differentiation, treatment of mouse and avian NCC with BMP-2, -4, or -7 *in vitro* led to increased levels of early SA markers MASH1 (ASCL1) and PHOX2B^42^,^52^,^53^,^55^. In our human PSC-derived trunk NCC, treatment with BMP-2, -4, or -7 stimulated expression of both early and late SA markers (Fig. 4A,B). Treatment with BMPs did not increase the expression of *DBH* mRNA in our studies, perhaps due to the limited time these cells were cultured prior to BMP treatment. Cells may be more responsive to BMP at longer time points, as DBH is among the latest of SA markers.

Our results are the first, to our knowledge, that demonstrate differentiation of human PSC to subtype specific NCC, namely to trunk NCC (in the presence of RA), or cranial NCC (in the absence of RA). We also clarify that BMP blockade is not required for human PSC differentiation to NCC, while BMP stimulation of trunk NCC enhanced expression of some SA markers. These human PSC-derived trunk and cranial NCC can be analyzed further to better understand human NCC biology and to identify distinguishing features of each subtype, as well as species-specific differences in distinct NCC subtypes. Our human PSC-derived trunk NCC can be further differentiated to SA cells, offering a renewable human cell model to study SA cell biology, and for diseases derived from SA cells.

## Methods

### Ethics statement

WTC iPSC were obtained from a healthy adult as previously described^49^. Informed consent has been obtained from all subjects. All protocols used in this study were approved by Human Gamete, Embryo and Stem Cell Research Committee of the University of California, San Francisco Stem Cell Research Oversight Committee and the methods were carried out in accordance with the approved guidelines.

### Pluripotent stem cell maintenance

H1 ESC and WTC iPSC were maintained on GelTrex (Life Technologies) coated 6-well plates in mTeSR1 media (Stemcell Technologies). Cells were passaged using Accutase and plated in mTeSR1 with 2 uM of Thiazoviven (Stemcell Technologies).

### Differentiation to neural crest cells and sympathoadrenal cells

Studer NCC differentiation protocol was performed as previously described^30^. Dalton NCC differentiation protocol was performed as previously described^31^ with modifications. Briefly, SB431542 (Tocris) was used at 10 uM instead of 20 uM and the GSK3β inhibitor CHIR99021 (Tocris) was used at 3 uM instead of BIO. For Protocol #2 and #3, LDN-193189 (Tocris) was used at 500 nM.

Differentiation of human PSC to trunk NCC was performed using the modified Dalton protocol with Retinoic Acid (1 uM, Sigma Aldrich) added starting at Day 3 and maintained throughout the duration of the protocol (Day 11).

Differentiation of trunk NCC to sympathoadrenal cells used the modified media from the Dalton protocol with different BMPs (all from Peprotech) including BMP-2 (50 ng/mL), BMP-4 (50 ng/mL), or BMP-7 (100 ng/mL) for 2 weeks.

### RT-qPCR

RNA was extracted using an RNA extraction kit (Zymo Research) and cDNA synthesis was performed with vilo (Life Technologies). qPCR was performed using SYBR green (KAPA Biosystems) on an AB7900HT. Gene expression was normalized to GAPDH and represented as fold increase over control cell lines.

### Antibodies

Primary antibodies for immunofluorescence were obtained commercially for HNK1 (Sigma Adlrich), p75 (Advance Targeting Systems), FOXD3 (R&D Systems), SOX10 (R&D Systems), PHOX2B (Proteintech), DBH (Abgent) and TH (R&D Systems). Secondary antibodies were obtained from Life Technologies. Coverslips with stained cells were mounted in Vectashield Hard Set containing DAPI (Vector Laboratories).

### Statistical Analysis

All gene expression data presented are averages of at least three experiments. *P* values were generated by one-tailed *t* test (unequal variance).

## Additional Information

**How to cite this article**: Huang, M. *et al.* Generating trunk neural crest from human pluripotent stem cells. *Sci. Rep.* **6**, 19727; doi: 10.1038/srep19727 (2016).

## Supplementary Material

Supplementary Information
