# Supplementary material for: Generating trunk neural crest from human pluripotent stem cells
Source: Sci Rep. 2016 Jan 27;6:19727. doi: 10.1038/srep19727 (PMC4728437; doi:10.1038/srep19727)
Supplement: Supplementary Information [file srep19727-s1.pdf]

Supplementary Information

Generating trunk neural crest from human pluripotent stem cells

Miller Huang[1], Matthew L Miller[1], Lauren K McHenry[1], Tina Zheng[1], Qiqi Zhen[1], Shirin Ilkhanizadeh[1], Bruce R Conklin[3,4], Marianne E Bronner[5] and William A Weiss[1,2,*]

[1]Department of Neurology and the Helen Diller Family Comprehensive Cancer Center, University of California, San Francisco, CA 94158 USA

[2]Departments of Pediatrics, Neurosurgery and Brain Tumor Research Center, University of California, San Francisco, CA 94158 USA

[3]Gladstone Institute of Cardiovascular Disease, San Francisco, CA 94158 USA

[4]Departments of Medicine and Cellular and Molecular Pharmacology, University of California, San Francisco, San Francisco, California 94143, USA

[5]Division of Biology and Biological Engineering, California Institute of Technology, Pasadena, California 91125, USA

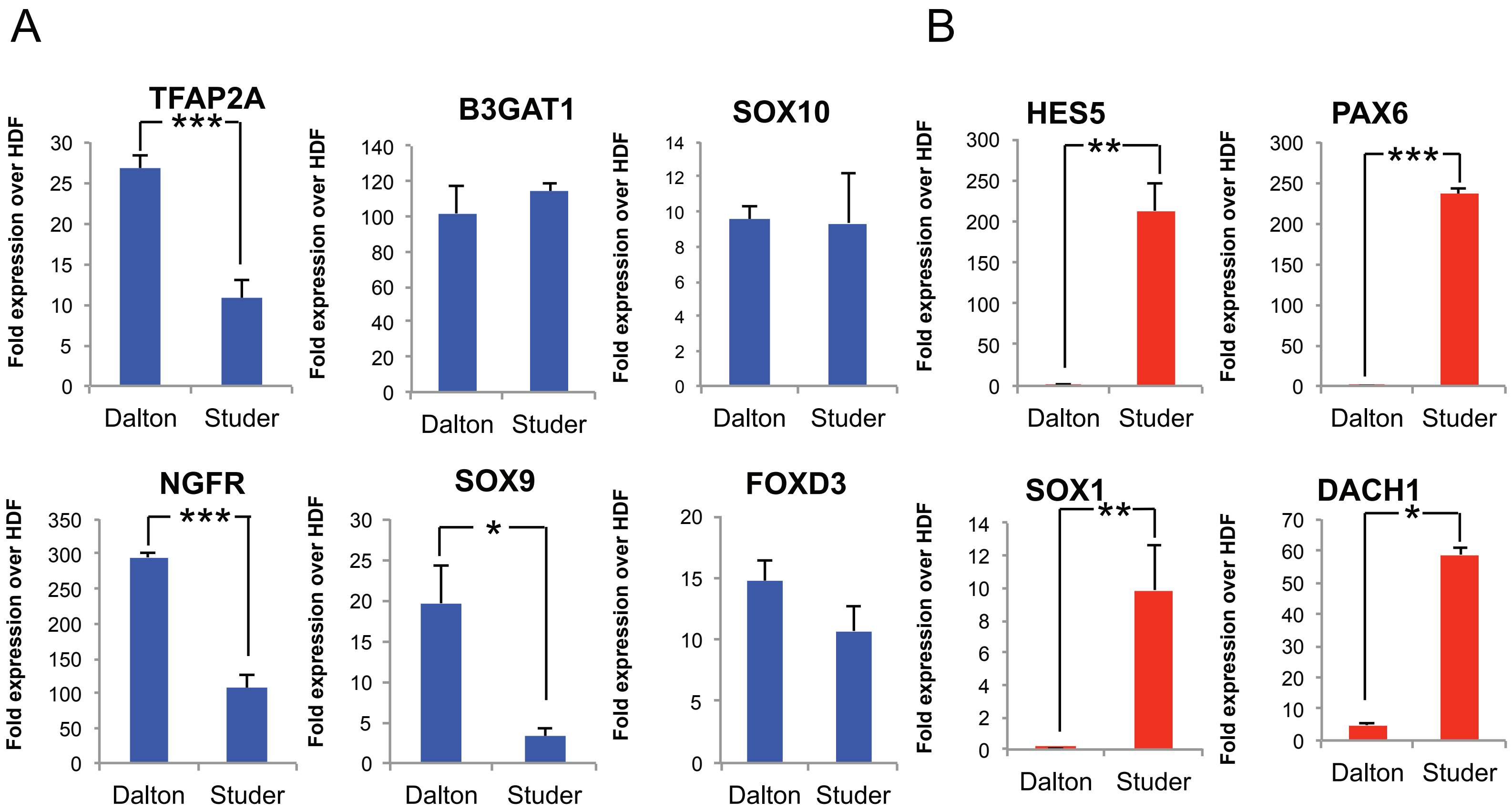

A
B
TFAP2A
***
B3GAT1
SOX10
HES5
**
PAX6
***
NGFR
***
SOX9
*
FOXD3
SOX1
**
DACH1
*
Fold expression over HDF
Dalton
Studer

Supplementary Figure S1: WTC iPSC was differentiated toward NCC via protocols published by the Dalton or Studer lab. At the end of each protocol, expression of **(A)** NCC markers and **(B)** CNS-related markers were analyzed using RT-qPCR and compared against the expression of these markers in human dermal fibroblasts (HDF). Dalton protocol showed higher levels of NCC markers, while suppressing CNS markers compared to the Studer protocol. *$p < 0.05$, **$p < 0.01$, ***$p < 0.001$.

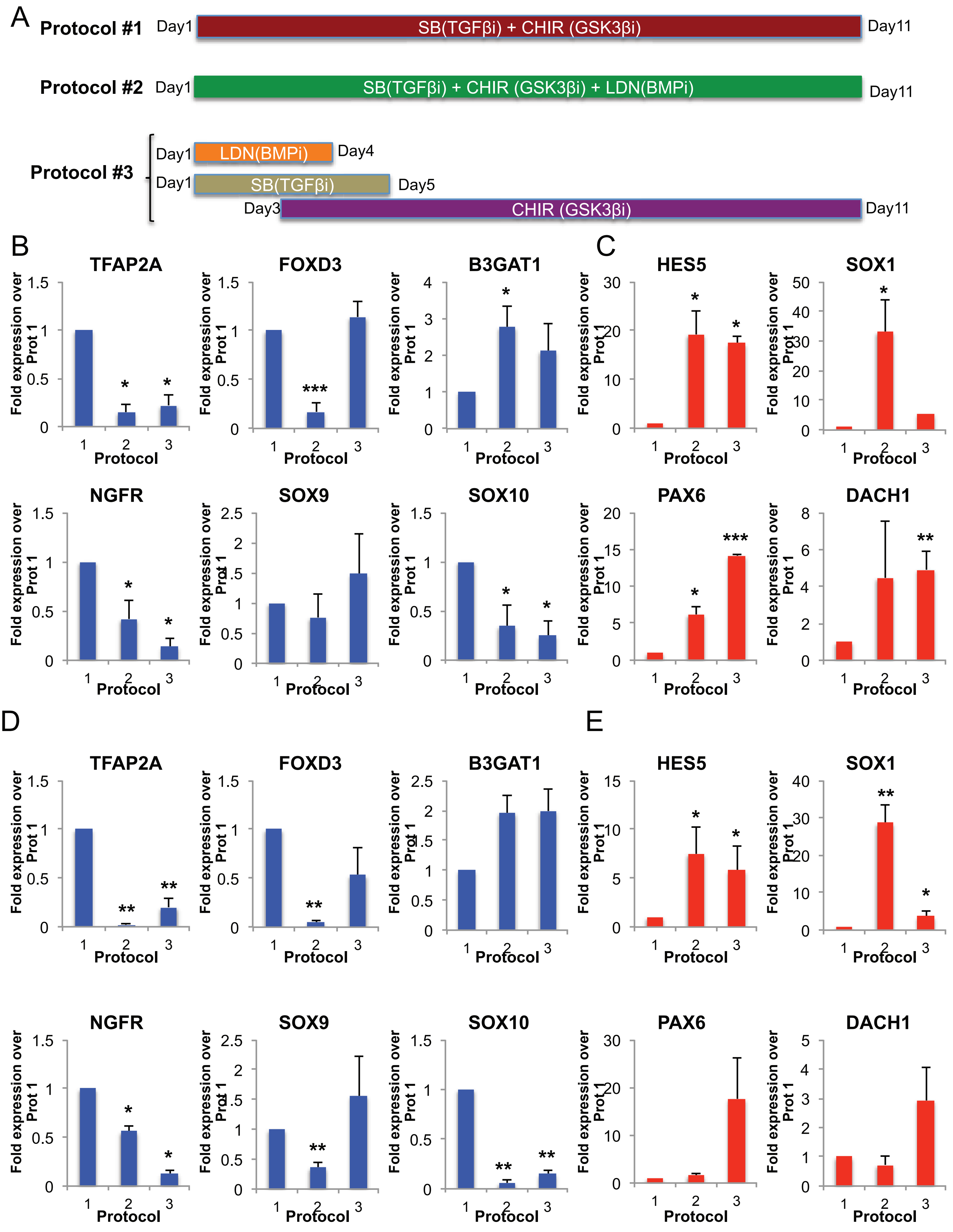
A
Protocol #1
Day1
SB(TGFβi) + CHIR (GSK3βi)
Day11
Protocol #2
Day1
SB(TGFβi) + CHIR (GSK3βi) + LDN(BMPi)
Day11
Protocol #3
Day1
LDN(BMPi)
Day4
Day1
SB(TGFβi)
Day5
Day3
CHIR (GSK3βi)
Day11
B
TFAP2A
FOXD3
B3GAT1
NGFR
SOX9
SOX10
C
HES5
SOX1
PAX6
DACH1
D
TFAP2A
FOXD3
B3GAT1
NGFR
SOX9
SOX10
E
HES5
SOX1
PAX6
DACH1
Fold expression over Prot 1
Protocol

Supplementary Figure S2: (A) Schematic of 3 different optimizations of NCC differentiation. Protocol #1 is the original Dalton protocol using SB431542 and CHIR99021 only. Protocol #2 includes the addition of the BMP inhibitor LDN193189. Protocol #3 follows the same timeline of inhibitor treatment as in the Studer protocol. **(B, C)** H1 ESC and **(D, E)** WTC iPSC were differentiated toward NCC via all 3 protocols. At the end of each protocol, expression of NCC markers **(B, D)** or CNS-related markers **(C, E)** was analyzed using RT-qPCR and compared against the expression of these markers using the original Dalton protocol (Protocol #1). *$p < 0.05$, **$p < 0.01$, ***$p < 0.001$.

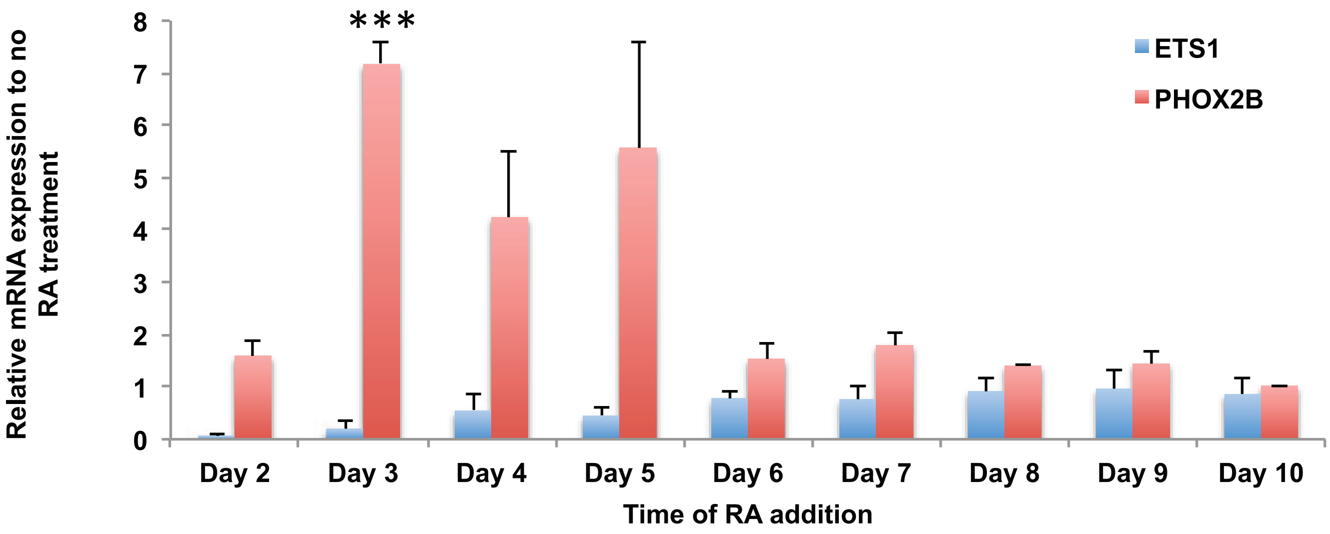

Relative mRNA expression to no RA treatment
8
7
6
5
4
3
2
1
0
***
ETS1
PHOX2B
Day 2
Day 3
Day 4
Day 5
Day 6
Day 7
Day 8
Day 9
Day 10
Time of RA addition

Supplementary Figure S3. RT-qPCR analysis for cranial (*ETS1*) and trunk (*PHOX2B*) markers from WTC cells differentiated with RA added starting at each day indicated, demonstrating that RA addition at Day 3, 4 or 5 resulted in maximal upregulation of trunk (*PHOX2B*) and suppression of cranial (*ETS1*) markers. ***p < 0.001
